# The Anti-Oxidant Ergothioneine Augments the Immunomodulatory Function of TLR Agonists by Direct Action on Macrophages

**DOI:** 10.1371/journal.pone.0169360

**Published:** 2017-01-23

**Authors:** Sumito Yoshida, Hiroaki Shime, Kenji Funami, Hiromi Takaki, Misako Matsumoto, Masanori Kasahara, Tsukasa Seya

**Affiliations:** 1 Department of Microbiology and Immunology, Graduate School of Medicine, Hokkaido University, Kita-ku, Sapporo City, Japan; 2 Department of Pathology I, Graduate School of Medicine, Hokkaido University, Kita-ku, Sapporo City, Japan; University of Torino, ITALY

## Abstract

L-Ergothioneine (EGT) is a naturally-occurring amino acid which is characterized by its antioxidant property; yet, the physiological role of EGT has yet to be established. We investigated the immune-enhancing properties of EGT, and found that it acts as a potentiator of toll-like receptor (TLR) signaling. When mouse bone marrow-derived macrophages (BMDMs) were pretreated with EGT, TLR signal-mediated cytokine production was augmented in BMDMs. The results were reproducible with TLR2, 3, 4 and 7 agonists. In particular, IL-6 and IL-12p40 were elevated further by pretreatment with EGT in BMDMs, suggesting the induction of M1 polarization. In co-culture assay with OT-II CD4^+^ T cells and splenic F4/80^+^ macrophages, EGT significantly induced Th17 skewing in CD4^+^ T cells. Thus, EGT is an immune modifier as well as a redox controller under TLR stimulation that induces M1 macrophages and a Th17 shift in inflammation.

## Introduction

Macrophages maintain immune homeostasis in response to extra-cellular conditions and regulate innate and cell-mediated immunity to promote cytokine networking, inflammation and tissue reorganization [1]. Macrophages, when activated in response to microbial patterns, quickly eliminate microbes by phagocytosis and production of ROS (reactive oxygen species). Two activation states are defined as M1 and M2 polarization in macrophages [2]. M1 macrophages produce proinflammatory cytokines such as IL-12, IL-6, IL-1β, and TNF-α, leading to induction of the Th1-type immune response and expression of high levels of inducible NO synthase (iNOS) that facilitates nitric oxide (NO) production. *In vitro* experiments have shown that TNF-α and NO produced by M1 macrophages are involved in direct killing of infected cells. On the other hand, M2 macrophages are characterized by higher expression levels of IL-10, arginase-1, scavenger receptor (SR) and mannose receptor (MMR, CD206), with lower expression of proinflammatory cytokines compared to M1-types.

Macrophage polarization is affected by several factors in the surrounding microenvironment [2,3]. The profiles of cytokines and mediators in macrophages are modulated by signals elicited by soluble factors or pattern-recognition receptors in infectious conditions. Th2 cytokines such as IL-4 and IL-13, transforming growth factor-β (TGF-β), prostaglandin E2 (PGE2), or hypoxia drives the development of M2-polarized macrophages. On the other hand, infections are usually accompanied by stimulation of TLR ligands such as lipopolysaccharide (LPS) which induce M1-polarization of macrophages.

L-Ergothioneine (EGT) is a thiol-containing antioxidant amino acid at physiological pH. Unlike other antioxidants such as glutathione or N-acetyl-L-cysteine (NAC, a precursor of glutathione), EGT is characterized by its slow degradation and resistance to disulfide formation [4,5]. EGT disulfide formation occurs only at low pH in the presence of Cu^++^ or H_2_O_2_, but not under physiological conditions. EGT acts as a cation chelator [6], bioenergetics factor [7], immune regulator [8,9] and antioxidant [10,11]. EGT is widely distributed within organs and in blood cells [12]. The tissue levels of expression of the EGT transporter, OCTN1 encoded by the *SLC22A4* gene, appear critical for EGT uptake [13]. OCTN1 is an integral membrane protein which transports EGT depending on the concentration of Na^+^ and H^+^ [14]. EGT also accumulates in the mitochondria [15,16] where OCTN1 is expressed, suggesting that EGT protects mitochondrial DNA and other constituents from damage by ROS generated through inflammation. However, the immune modulatory function of EGT remains largely uncharacterized.

In the present study, we investigated the function of EGT in mouse macrophages following stimulation with TLR agonists. We found that pretreatment of macrophages with EGT augments cytokine production induced by TLR ligands and enhances Th17 polarization of CD4^+^ T cells.

## Materials and Methods

### Mice

Inbred C57BL/6 wild-type (female B6 WT) mice were purchased from Clea Japan. OT-II T cell receptor (TCR) transgenic mice (OT-II mice) were kindly provided by Dr. K. Iwabuchi (Kitasato University). Female mice of 6–10 week of age were used in all experiments and were maintained under specific pathogen-free conditions. The protocol was approved by the Committee on the Ethics of Animal Experiments in the Animal Safety Center, Hokkaido University Japan. All mice were used according to the guidelines of the institutional animal care and use committee of Hokkaido University.

### Cell isolation and culture

Cells were cultured at 37°C under 5% CO_2_ in RPMI1640 medium supplemented with 10% heat-inactivated fetal bovine serum (FBS), 1 mM hydroxyethyl-piperazine ethanesulafonic acid (HEPES), 55 μM 2-mercaptoethanol (2-ME), 100 mU/mL penicillin and 100 μg/mL streptomycin. For preparation of bone marrow-derived macrophages (BMDMs), bone marrow cells from B6 mice were cultured in RPMI1640 supplemented with 10% FBS, 100 mU/mL penicillin and 100 μg/mL streptomycin, and 30% L929 cell conditioned medium for 6 days. Medium was exchanged every 3 days with fresh one. F4/80^+^ cells were isolated from single cell suspension of collagenase D (Roche, Mahnheim, Germany)-treated spleens of B6 mice, and stained with biotin-conjugated anti-F4/80 antibody (Ab) (BM8; Biolegend, San-Diego, CA) and streptavidin microbeads (Miltenyi Biotec, Bergisch Gladbach, Germany). Isolation of CD11c^+^ cells from B6 mouse spleens and CD4^+^ cells from OT-II mouse spleens was performed using anti-CD11c Ab-conjugated microbeads and anti-CD4 Ab-conjugated microbeads (Miltenyi Biotec), respectively. All chemicals were special grades for cell culture.

Cells were cultured in the medium in the presence of 1~10 mM of L-ergothioneine (EGT) (TETRAHEDRON, Paris, France) for 24 hours and then stimulated with indicated amounts of Pam2CSK4 (CS Bio, Shanghai, China), Pam3CSK4 (Boehringer Mannheim, Mannheim, Germany), polyinosine-polycytidylic acid (poly I:C) (GE healthcare, Piscataway, NJ), lipopolysaccharide (LPS) (Sigma-Aldrich, St Louis, MO), or gardiquimod (Invivogen, San-Diego, CA). After Twenty-four hours, cytokine concentrations in the conditioned medium were determined by cytometric beads assay (CBA) or ELISA according to the manufacturer’s instructions.

Co-culture experiments were performed in a 24 well-plate as described previously [17]. Briefly, F4/80^+^ cells (1 x 10^5^/well) or CD11c^+^ cells (0.5 x 10^5^/well) were co-cultured with CD4^+^ OT-II T cells at 1:1 ratio in the presence of 0.1 μg/mL ovalbumin (OVA)_323-339_ peptide in 1mL medium. After Eighty-four hours, culture supernatant was used for CBA assay or ELISA. For intracellular cytokine staining of CD4^+^ T cells, cells were stimulated with 50 ng/mL phorbol 12-myristate 13-acetate (PMA) and 750 ng/mL ionomycin for 5 hours in the presence of 10 μg/mL Brefeldin A [18]. Single cell suspension was incubated with anti-CD16/32 Ab (2.4G2) for 10 min. to block FcγR and then stained with PE-anti-CD3ε Ab (145-2C11) and PerCP-anti-CD4 Ab (RM4-5) for 20 min. Cells were fixed, permeabilized by BD Cytofix/Cytoperm (BD Bioscience, San Jose, CA) and stained with APC-anti-IL-17A Ab (TC11-18H10.1) and FITC-anti-IFN-γ Ab (XMG1.2) for 30 min. Flow cytometric analysis was performed on the FACS Caliber (BD Bioscience) or the FACS Aria II (BD Bioscience) and analyzed by FlowJo software (TreeStar, Ashland, OR). All fluorescence-labeled antibodies were purchased from BioLegend (San-Diego, CA).

### Measurement of intracellular reactive oxygen species (ROS)

BMDMs (1 x 10^5^ cells/well) were suspended in phenol red-free RPMI1640 supplemented with 1% FBS and 10% L929 cell conditioned medium, and cultured in the presence of EGT, *N*-acetyl-L-cysteine (NAC; Sigma-Aldrich), or gluthatione (GSH; Sigma-Aldrich). After 2 or 24 hours, BMDMs were stimulated with 100 ng/mL LPS for 4 hours and then stained with 2.5 μM CM-H_2_DCFDA (5-(and-6)-chloromethyl-2´,7´-dichlorodihydrofluorescein diacetate) (Invitrogen, Carlsbad, CA) for 30 min. The fluorescence was determined with Trister LB941 (Berthold Technologies, Pforzheim, Germany).

### Quantitative RT-PCR

Total RNA was isolated from cells stimulated with TLR ligands using RNeasy Mini prep kit (QIAGEN, Nilden, Germany). cDNA was synthesized with High-capacity cDNA Reverse Transcription Kit (Applied Biosystems, Foster, CA) using random hexamers as described in the manufacturer's instructions. Quantitative PCR analysis was performed with Power SYBR Green PCR Master Mix (Applied Biosystems) and StepOne Real-Time PCR system (Applied Biosystems). Expression of the cytokine genes was normalized to the expression of mouse GAPDH. We used following primer pairs: mouse GAPDH forward, 5’-GCCTGGAGAAACCTGCCA-3’; mouse GAPDH reverse, 5’-CCCTCAGATGCCTGCTTCA-3’; mouse IL-12p35 forward, 5’-ATGTGTCTCCCAAGGTCAGC-3’; mouse IL-12p35 reverse, 5’-ATGACCCTGGCCAAACTGAG-3’; mouse IL-12p40 forward, 5’-AATGTCTGCGTGCAAGCTCA-3’; mouse IL-12p40 reverse, 5’-ATGCCCACTTGCTGCATGA-3’; mouse IL-23p19 forward, 5’-TCTGCATGCTAGCCTGGAAC-3’; mouse IL-23p19 reverse, 5’-TGGCTGTTGTCCTTGAGTCC-3’; mouse IL-6 forward, 5’-GTTCTCTGGGAAATCGTGGA-3’; mouse IL-6 reverse, 5’-TCCAGTTTGGTAGCATCCATC-3’; mouse IL-10 forward, 5’- GGCGCTGTCATCGATTTCTC-3’; mouse IL-10 reverse, 5’-TGCTCCACTGCCTTGCTCTTA-3’; mouse IL-1β forward, 5’- TGACGGACCCCAAAAGATGA-3’; mouse IL-1β reverse 5’-TGCTGCTGCGAGATTTGAAG-3’; mouse OCTN1 forward, 5’-TACGAAGAACAGGGAGGTGG-3’; mouse OCTN1 reverse, 5’-GCTGGGAGTACGACAAGGAC-3’; mouse ASCT2 forward, 5’-GGACGTCTTTCATCTCCACAA-3’; mouse ASCT2 reverse, 5’-ACTCCTTCAATGATGCCACC-3’; mouse Arg-1 forward, 5’-GGAATCTGCATGGGCAACCTGTGT-3’; mouse Arg-1 reverse, 5’-AGGGTCTACGTCTCGCAAGCCA-3’; mouse Chi3l3 forward, 5’-TCACTTACACACATGAGCAAGAC-3’; mouse Chi3l3 reverse, 5’-CGGTTCTGAGGAGTAGAGACCA-3’; mouse Retnla forward, 5’-CCAATCCAGCTAACTATCCCTCC-3’; mouse Retnla reverse, 5’-ACCCAGTAGCAGTCATCCCA-3’. Data were analyzed by the ΔΔCt method.

### Statistical analysis

**Fig 1:**
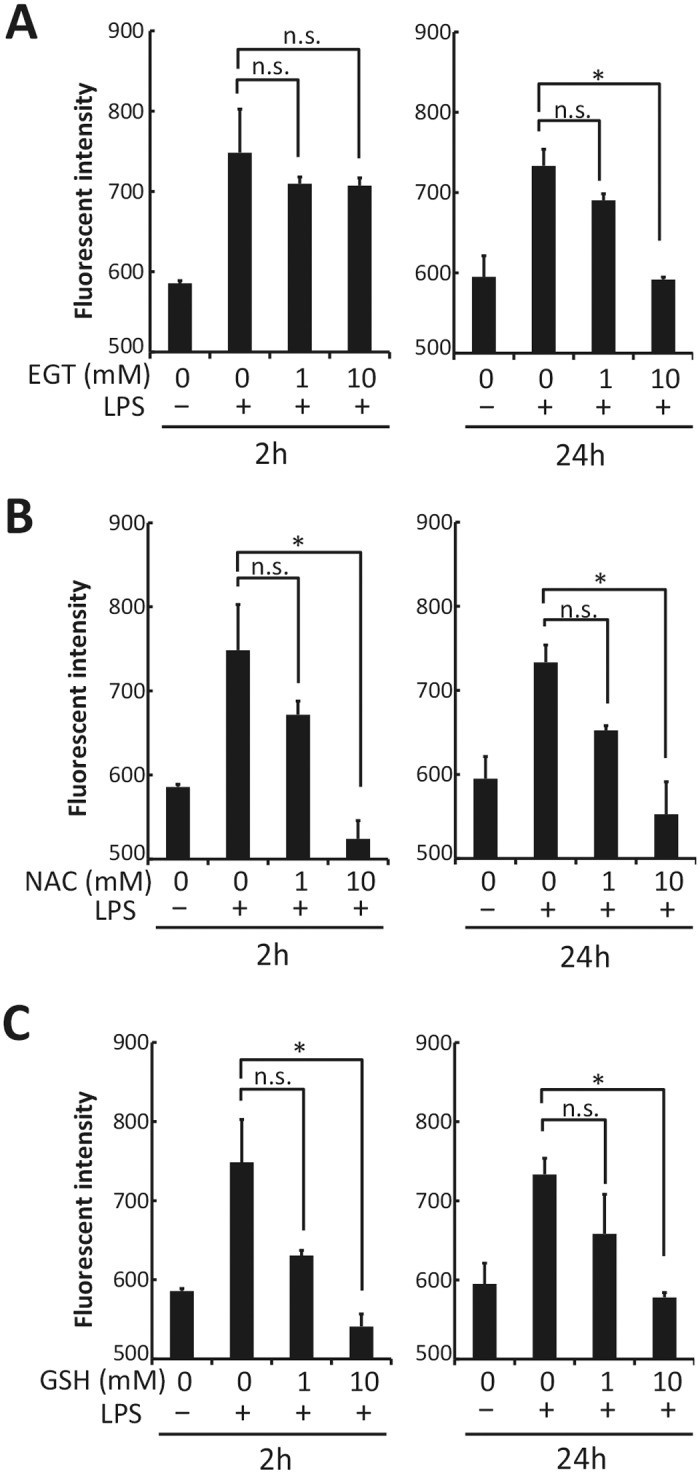
EGT scavenges intracellular ROS. BMDMs were pretreated with PBS, 1 or 10 mM EGT (A), 1 or 10 mM NAC (B), or 1 or 10 mM GSH (C) for 2 or 24 hours, and then were stimulated with 100 ng/mL LPS for 4 hours. Intracellular ROS was measured with CM-H_2_DCFDA as described in Materials and Methods. *n* = 3. Data are shown as mean ± SD. *P < 0.05. n.s, not significant.

The statistical significance of the obtained data in this study was analyzed using a two tail unpaired *t* test except for Fig 1, where one-way ANOVA with Bonferroni multiple comparisons test was employed for post-hoc comparisons. In either case, *p* < 0.05 was regarded as statistically significant.

## Results

### EGT scavenges ROS generated in macrophages after LPS stimulation

Bone marrow-derived macrophages (BMDMs) were stimulated with LPS, and intracellular production of ROS was observed as reported previously [18,19]. Pre-treatment of BMDMs with 10 mM EGT for 24 h resulted in abrogation of LPS-induced ROS production (Fig 1A), while a lower concentration (1 mM) of EGT or shorter preincubation period (2 h) was insufficient for BMDMs to block ROS production (Fig 1A). In contrast, preincubation for 2 h was sufficient for the other ROS scavengers, NAC (Fig 1B) and glutathione (Fig 1C) to inhibit ROS production triggered by LPS stimulation.

### EGT acts as an enhancer of TLR ligand-induced cytokine production

**Fig 2:**
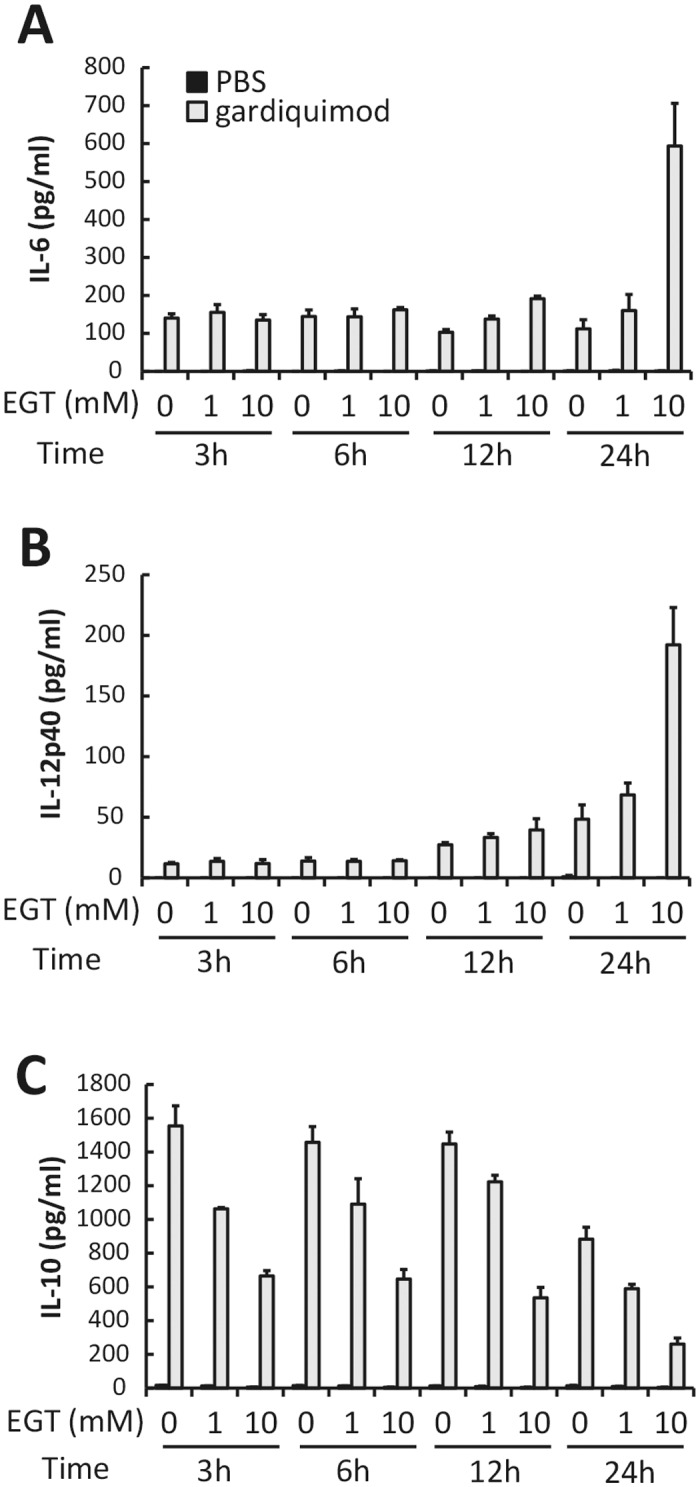
Preincubation is required for EGT-induced enhancement of cytokine response of BMDMs to TLR ligand. BMDMs pretreated with PBS or EGT (1 or 10 mM) for indicated time were stimulated with 1 μg/mL gardiquimod for 24 hours. Concentration of IL-6 (A), IL-12p40 (B), and IL-10 (C) in the conditioned media was determined. *n* = 3. Data are shown as mean ± SD.

EGT affects cell survival and plays a part in inhibition of JNK-mediated IL-6 [8] and TNF-α-mediated IL-8 production [9]. EGT appears to suppress immune cell activation by acting on mitochondria in conjunction with its anti-oxidant function. In fact, EGT had no effect on cytokine production from BMDMs (Fig 2A, 2B and 2C). However, EGT unexpectedly modulated cytokine production in response to inflammatory conditions such as stimulation with TLR agonists. When BMDMs were stimulated with gardiquimod, a TLR7 ligand, they produced IL-6 and IL-12p40 and this response was augmented by pre-incubation with EGT for 24 h (Fig 2A and 2B). Interestingly, Preincubation time less than 12 h was insufficient for this response. In contrast, less IL-10 was produced in EGT-pretreated BMDMs compared with untreated BMDMs in response to gardiquimod stimulation, in which 2 h pretreatment with EGT was sufficient (Fig 2C). These results suggest that EGT acts as a modulator of cytokine production in macrophages over TLR ligand stimulation.

**Fig 3:**
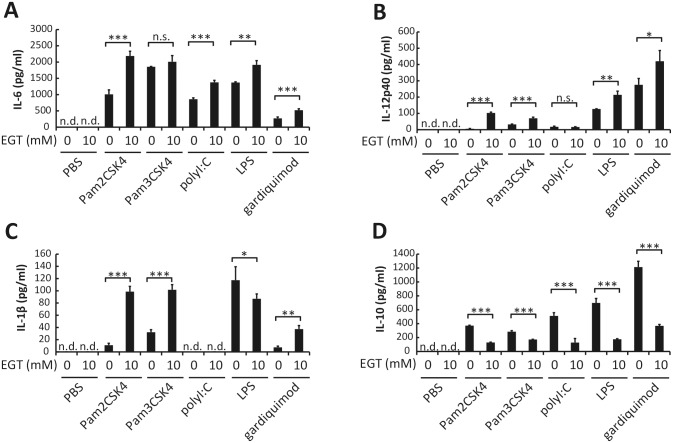
EGT enhances production of proinflammatory cytokines by BMDMs stimulated with TLR ligands. BMDMs pretreated with PBS or 10 mM EGT for 24 hours were stimulated with 50 nM Pam2CSK4, 100 ng/mL Pam3SCK4, 25 μg/mL poly I:C, 100 ng/mL LPS, or 1 μg/mL gardiquimod for 24 hours. Concentration of IL-6 (A), IL-12p40 (B), IL-1β (C), and IL-10 (D) in the conditioned media was determined. *n* = 3. Data are shown as mean ± SD. *P < 0.05, **P < 0.005, **P < 0.0005. n.s., not significant. n.d., not detected.

**Fig 4:**
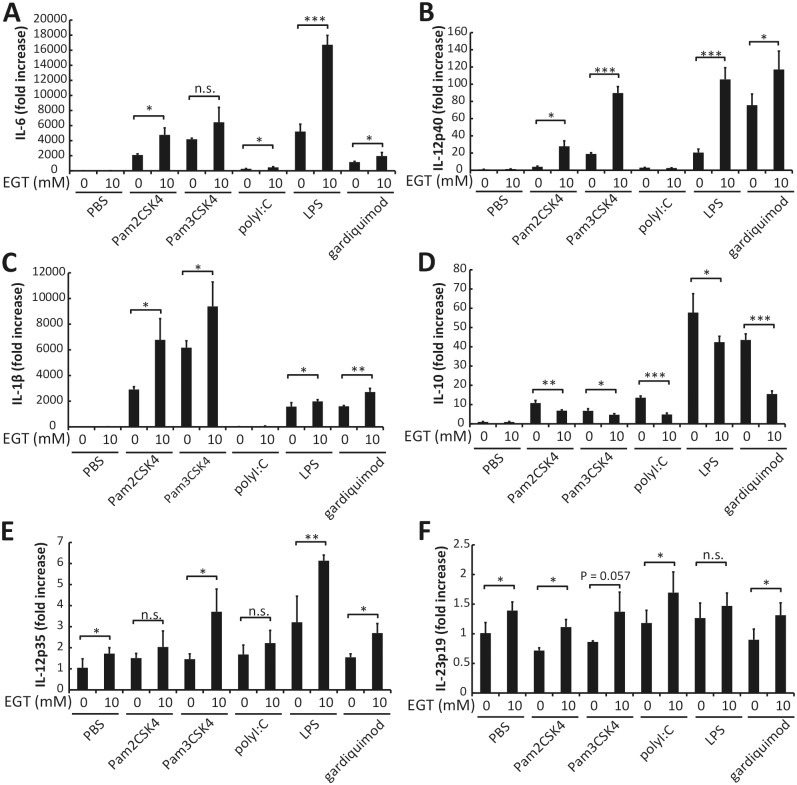
EGT enhances gene expression of cytokines in BMDMs stimulated with TLR ligands. BMDMs pretreated with PBS or 10 mM EGT for 24 hours were stimulated with 50 nM Pam2CSK4, 100 ng/mL Pam3CSK4, 25 μg/mL1, 100 ng/mL LPS, or 1 μg/mL gardiquimod for 4 hours. mRNA expression of IL-6 (A), IL-12p40 (B), IL-1β (C), IL-10 (D), IL-12p35 (E), and IL-23p19 (F) in BMDMs was determined. *n* = 3. Data are shown as mean ± SD. *P < 0.05, **P < 0.005, ***P < 0.0005. n.s., not significant.

We further examined the effects of EGT on cytokine production from BMDMs following stimulation with other TLR agonists. Pam2CSK4 (TLR2/6 agonist), Pam3CSK4 (TLR2/1 agonist), and gardiquimod all induced production of IL-6, IL-12p40, IL-1β, and IL-10 from BMDMs. Poly I:C (TLR3 agonist) induced production of IL-6, IL-12p40, and IL-10, but not IL-1β. Pretreatment of BMDMs with 10 mM EGT for 24 h up-regulated the production of IL-6 induced by all of the TLR agonists except for Pam3CSK4 (Fig 3A). Production of IL-12p40 and IL-1β, which was induced by all of the TLR ligands except for poly I:C, was also up-regulated in BMDMs pretreated with EGT (Fig 3B and 3C). In contrast, TLR ligand-induced IL-10 production was down-regulated in BMDMs by pretreatment with EGT (Fig 3D). Similar effects of EGT were observed when mRNA levels of these cytokines were analyzed (Fig 4). EGT treatment barely affected cell numbers and dead cell ratio of macrophages (S1 Fig), but M2 markers such as Arg-1 and CD206 are reduced in response to EGT and TLR agonists (S2 Fig). The results infer that EGT promotes M1 polarization on TLR stimuli in macrophages; yet no significant cytokine response occurs unless TLR stimulation is added to EGT treatment in macrophages.

Since IL-6 and IL-12p40 production induced by TLR signaling was enhanced in the presence of EGT, it raised the possibility that Th1 and Th17 skewing by macrophages are influenced by EGT. This idea was supported by data showing that mRNA expression of IL-12 p35 and IL-23 p19 was also up-regulated by EGT pretreatment.

### EGT enhances IL-17 but not IFN-γ production in F4/80^+^ macrophage–T cell co-culture

**Fig 5:**
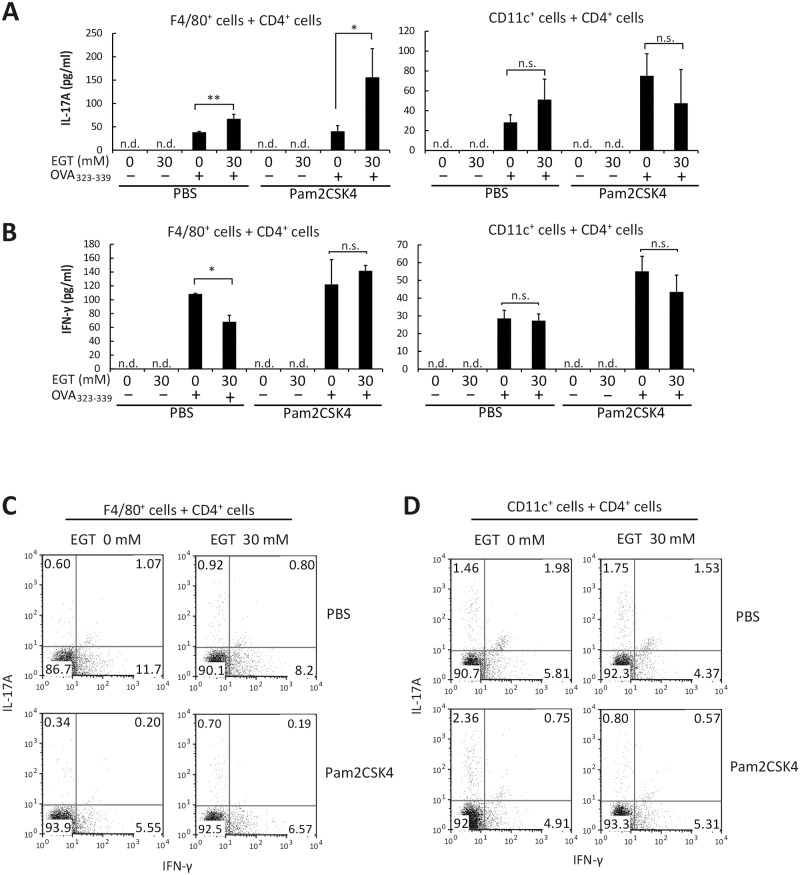
EGT enhances Th17 induction by splenic F4/80^+^ cells but not CD11c^+^ cells. F4/80^+^ cells or CD11c^+^ cells isolated from B6 mouse spleen were treated with PBS or 30 mM EGT for 24 hours. CD4^+^ OT-II T cells were mixed with PBS or EGT-treated F4/80^+^ cells or CD11c^+^ cells. The mixture was incubated for 84 hours in the presence or absence of 50 nM Pam2CSK4. Concentration of IL-17A (A) or IFN-γ (B) in the conditioned media was determined and intracellular cytokine staining of IL-17A and IFN-γ in CD4^+^ T cells was performed (C, D). *n* = 3. Data are shown as average ± SD. *P < 0.05, **P < 0.005. n.s., not significant. n.d., not detected.

To test whether EGT enhances antigen (Ag)-dependent Th17 polarization under Pam2CSK4 stimulation, F4/80^+^ cells were isolated from spleen and pretreated with EGT for 24 h. Then, the F4/80^+^ cells were co-cultured with OT-II CD4^+^ T cells in the presence of antigen peptide (OVA_323-339_) and TLR ligand (Pam2CSK4). Without antigen, no T cell proliferative response occurred. A minimal T cell response was observed with Pam2CSK4 only, while a strong response is observed with antigen and TLR agonist in OT-II cells [20]. No such OVA-specific T cell response was observed in WT spleen cells, which had not been sensitized with OVA. Under these conditions, IL-17A and IFN-γ were measured by CBA assay (Fig 5A and 5B). EGT (30 mM) pretreatment resulted in enhanced IL-17A production in the conditioned medium in response to Pam2CSK4 and antigen (Fig 5A). EGT increased the population of IL-17-producing cells in the co-culture assay using F4/80^+^ and CD4^+^ T cells (Fig 5C). IFN-γ production was also induced by Pam2CSK4 and antigen, but not enhanced by pretreatment of F4/80^+^ cells with EGT (Fig 5B). EGT barely affected Th17 differentiation of CD4^+^ T cells induced by CD11c^+^ cells compared to F4/80^+^ cells (right panels of Fig 5B).

**Fig 6:**
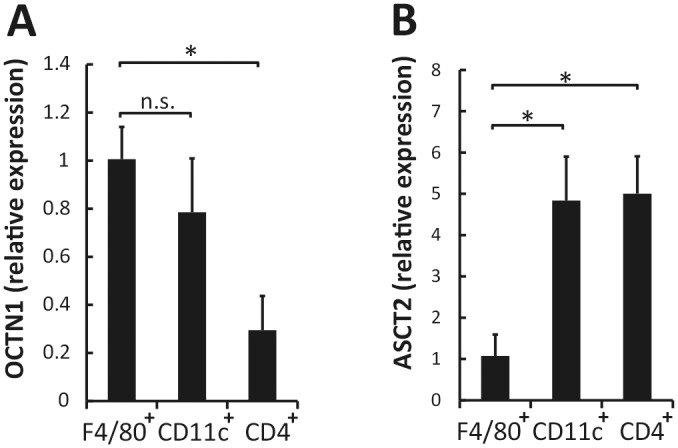
EGT transporter and NAC transporter are selectively expressed in immune cells. Total RNA was extracted from F4/80^+^ cells, CD11c^+^ cells, and CD4^+^ T cells to analyze gene expression of OCTN1 (A) and ASCT2 (B); n = 3. Data are shown as mean ± SD. *P < 0.05. n.s., not significant.

As shown in Fig 6A, OCTN1 is expressed in both of F4/80^+^ and CD11c^+^ cells. To clarify whether this effect of EGT is macrophage-specific, we co-cultured CD11c^+^ cells and CD4^+^ T cells and examined cytokine production. Interestingly, pretreatment of CD11c^+^ cells with EGT did not result in enhancement of IL-17A or IFN-γ production (Fig 5D).

### Enhancement of Th17 differentiation is a specific effect of EGT

**Fig 7:**
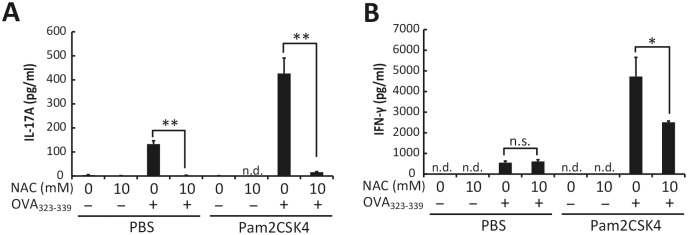
NAC does not enhance Pam2CSK4-induced IL-17 and IFN-γ production. F4/80^+^ cells isolated from B6 mouse spleen were treated with PBS or 10 mM NAC for 24 hours and then mixed with OT-II CD4^+^ T cells. The mixture was incubated for 84 hours in the presence or absence of 50 nM Pam2CSK4 and OVA_323-339_. Concentration of IL-17A (A) or IFN-γ (B) in the conditioned media was determined. *n* = 3. Data are shown as average ± SD. *P < 0.05, **P < 0.005. n.s., not significant. n.d., not detected.

To test whether the cytokine production and Th17 skewing are specific to EGT, we examined the effect of NAC on TLR agonist-induced cytokine production in BMDMs. The cytokine modulatory profile of EGT was shared with NAC in BMDMs (S3 Fig). In the presence of Pam2CSK4 stimulation, the profiles of cytokine production and Th17 induction of F4/80^+^ cells treated with EGT were compared with those treated with NAC. This showed that NAC inhibited, rather than enhanced, production of both IL-17 and IFN-γ (Fig 7A and 7B). To ensure that EGT and NAC acted intracellularly in those cells, we measured RNA expression of the EGT-specific transporter, OCTN1, and NAC transporter, ASCT2, in F4/80^+^ cells, CD4^+^ T cells, and CD11c^+^ cells. OCTN1 was highly expressed in F4/80^+^ cells and CD11c^+^ cells compared with CD4^+^ T cells whereas ASCT2 was preferentially expressed in CD4^+^ T cells and CD11c^+^ cells compared with F4/80^+^ cells (Fig 6A and 6B).

## Discussion

Here, we have first demonstrated the immune-activating function of EGT that is exerted under TLR stimulation. Murine macrophages express various TLRs and respond to pathogen-associated molecular patterns (PAMPs) to induce cytokines and modulate inflammation. Our finding was that proinflammatory cytokines including IL-6, IL-12p40, and IL-1β were markedly elevated by pre-EGT treatment upon PAMP stimulation while IL-10 was down-regulated in BMDMs pretreated with EGT. Hence, the BMDMs are susceptible to EGT which can skew activation to produce inflammatory or instructive cytokines. The cytokine production in BMDMs appears to occur in parallel with the ROS inhibitory function of EGT.

EGT significantly enhanced IL-6 and IL-12p40 production from BMDMs upon TLR ligand stimulation. IL-12p40 participates with p35 in the formation of IL-12p70 and with p19 in the formation of IL-23. PCR and ELISA analysis suggest that IL-23 is up-regulated in BMDMs in response to EGT and Pam2CSK4. IL-6 and IL-23 promote Th17 differentiation of naive CD4^+^ T cells [21]. We showed this is true in the EGT-treated macrophages prepared from mouse spleen. The *in vitro* results on BMDMs may be reflected in mouse models. Thus, Th17 polarization would be actually enhanced in mice treated with EGT under Pam2CSK4 stimulation. Since the degree of Th17 polarization induced by IL-23 closely links promotion of inflammation-based autoimmunity [22], EGT would be an endogenous factor for modulating autoimmune disorders.

A marked immunological finding in this study is that EGT specifically acts on macrophages to induce a Th17 shift in CD4^+^ T cells. This activity is unique to EGT because another antioxidant NAC lacks the ability to enhance Th17 polarization. Several reports suggest that both of these antioxidants are internalized into cells via transporter molecules [14,23]. OCTN1 is a transporter for EGT incorporation and distributed predominantly in macrophages, whereas ASCT2, a transporter for NAC, is ubiquitously distributed in blood cells including lymphocytes. It is therefore likely that the effects of EGT are restricted to macrophages because this cell-type preferentially expresses OCTN1: EGT are unlikely to be internalized into CD4^+^ T lymphocytes that minimally express OCTN1. Therefore, EGT-mediated functional modulation of macrophages directly induces a Th17 shift. On the other hand, NAC acts on CD4^+^ T cells, which probably leads to the inhibition of Th17 polarization of CD4 T^+^ cells by unknown mechanism. Interestingly, EGT did not enhance Th17 polarization induced by CD11c^+^ dendritic cells (DCs), suggesting that EGT-mediated up-regulation of cytokine response occurs specifically in macrophages but not CD11c^+^ DCs, although OCTN1 mRNA is expressed in CD11c^+^ cells at the comparable level to F4/80^+^ cells. The mechanism by which EGT endows the IL17-producing phenotype on CD4^+^ T cells induced by macrophages but not CD11c^+^ remains undetermined and further study is required.

Effect of antioxidant on the production of IL-6 and IL-12p40 is controversial. Alam et al. have shown that NAC-induced alteration of redox state affects IL-12 production in macrophages both positively and negatively through calmodulin and c-Rel [24]. Another group has reported that NAC treatment inhibits LPS-induced IL-6 production [25], and that IL-10 reciprocally suppresses IL-12 production in mouse macrophages [26]. The latter result would be consistent with our finding that EGT enhances expression of IL-12 while IL-10 is reduced in response to TLR stimulation. Additionally, the antigen presenting properties of macrophages appear to be kept unchanged in EGT treatment (data not shown). EGT might engage Th17 differentiation as well as cytokine network through epigenetic regulation in macrophages. At least, Epigenetic control is actually pivotal in differentiation in CD4^+^ T subsets, such as *Foxp3*^+^ regulatory T cells [27].

We demonstrate that pretreatment with EGT enhances transcription of M1-related cytokine genes induced by TLR ligands, suggesting that EGT-induced alteration of redox state positively regulates promoter activity of M1-related cytokine genes through transcription factors. Recent studies have demonstrated that M1/M2-polarization of macrophages is regulated by multiple signaling pathway [28]. IFN-γ and IFN-β, that are potent stimulators of macrophages, induce M1-like macrophages through JAK/STAT1 activation [29]. M1 macrophages derived from granulocyte macrophage colony-stimulating factor (GM-CSF)-treated human monocytes highly express IRF5 compared with macrophages derived through macrophage colony-stimulating factor (M-CSF) treatment. Overexpression of IRF5 in M2 macrophages forced them to differentiate into macrophages expressing M1-specific cytokines, leading to both Th1 and Th17 cell development [30]. Notably, a similar response was induced by EGT treatment following TLR stimulation of macrophages. Furthermore, several reports have suggested that TLR signals induce chromatin remodeling to control gene expression through transcription factors [31,32], which may regulate the expression levels of the transcription factors involved in macrophage polarization. Hence, changing the plasticity of macrophage function could be achieved by EGT and TLR signaling through epigenetic control of gene expression as suggested in several previous reports [28,33].

Our study on EGT acting as an immunological modifier sheds light on the new function of EGT, and demonstrates that TLR-induced cytokines are up-regulated by EGT. Thus, EGT is not just a thiol-containing amino acid which regulates the intracellular redox state but an immune cell modifier that augments TLR-mediated cytokine induction and Th17 skewing. In a tumor-bearing mouse model, CD11b^+^Ly6G^+^ cells (i.e. myeloid-derived suppressor cells) express high tumoricidal activity in response to TLR3 agonist by production of reactive oxygen species (ROS) [34]. As EGT is an antioxidant and naturally distributed in human blood and organs [4], *in vivo* immunological functions of EGT needs to be further addressed for future studies.

## Supporting Information

S1 Fig EGT barely affected viability of BMDMs. (A) BMDMs were plated into 96-well plate and treated with PBS or EGT for 3~24 hrs. Cell viability was assessed by WST-1 assay. (B) BMDMs were treated with PBS or EGT for 24 hrs and stained with propidium iodide (PI).
(TIF)

S2 Fig EGT reduced expression levels of some M2 markers of macrophages. (A) BMDMs were treated with PBS or EGT for 24 hrs and then stimulated with Pam2CSK4, Pam3CSK4, poly I:C, LPS or gardiquimod. After 4 hrs, total RNA was extracted from the cells and subjected to RT-qPCR. (B) BMDMs were treated with PBS or EGT and then stimulated with the TLR ligands as in panel A. After 24 hrs, CD206 expression levels on the cells were measured by FACS.
(TIF)

S3 Fig NAC enhances production of proinflammatory cytokines by BMDMs stimulated with TLR ligands. BMDMs were pretreated with PBS or NAC for 24 hrs, and then stimulated with the indicated TLR ligands for 24 hrs. The supernatant levels of (A) IL-6, (B) IL-12p40, (C) IL-1β, and (D) IL-10 were determined.
(TIF)
