# Supplementary figures and images for: The Anti-Oxidant Ergothioneine Augments the Immunomodulatory Function of TLR Agonists by Direct Action on Macrophages
Source: PLoS One. 2017 Jan 23;12(1):e0169360. doi: 10.1371/journal.pone.0169360 (PMC5256913; doi:10.1371/journal.pone.0169360)

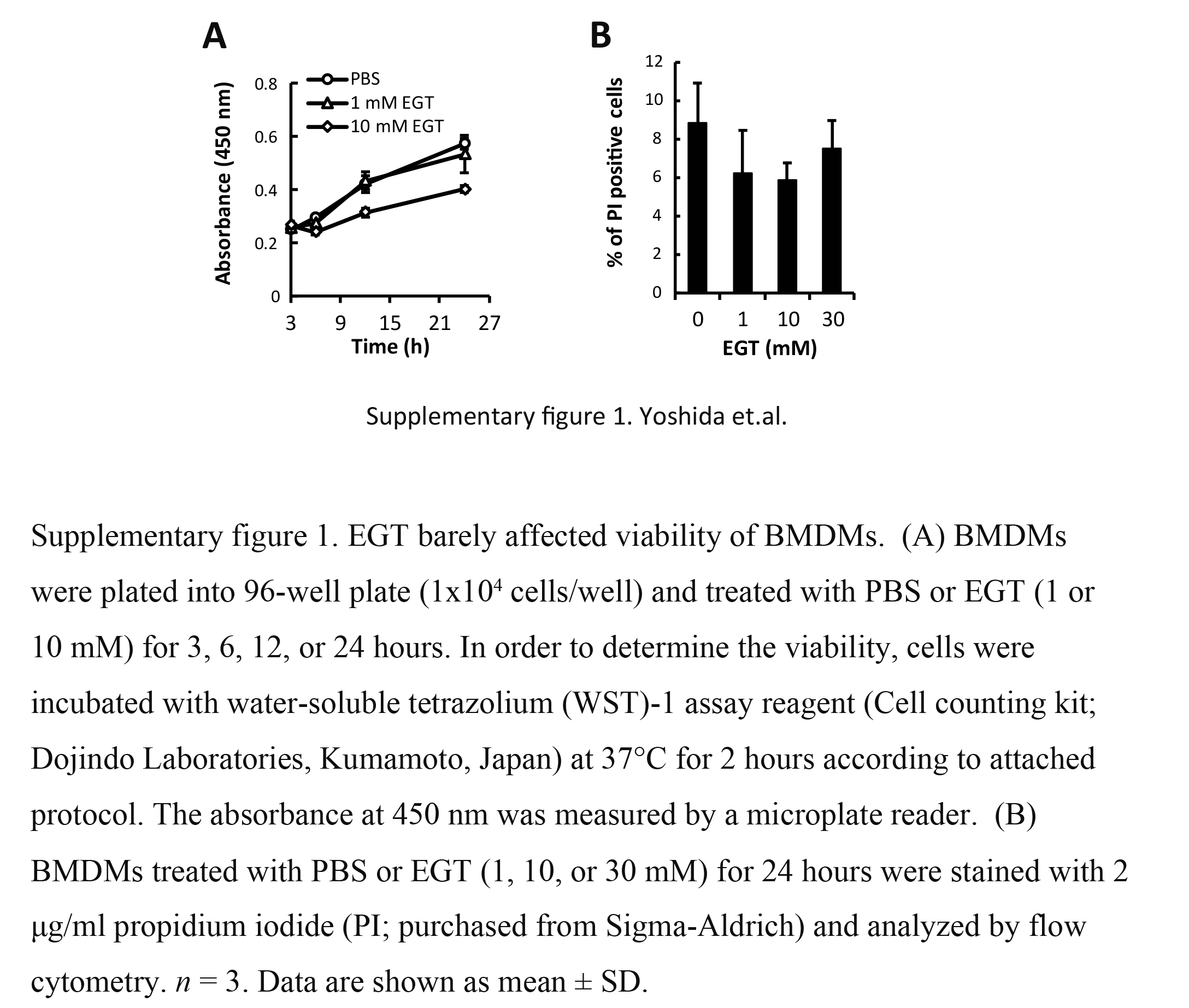

Supplement: S1 Fig — (A) BMDMs were plated into 96-well plate and treated with PBS or EGT for 3~24 hrs. Cell viability was assessed by WST-1 assay. (B) BMDMs were treated with PBS or EGT for 24 hrs and stained with propidium iodide (PI). (TIF) [file pone.0169360.s001.tif]

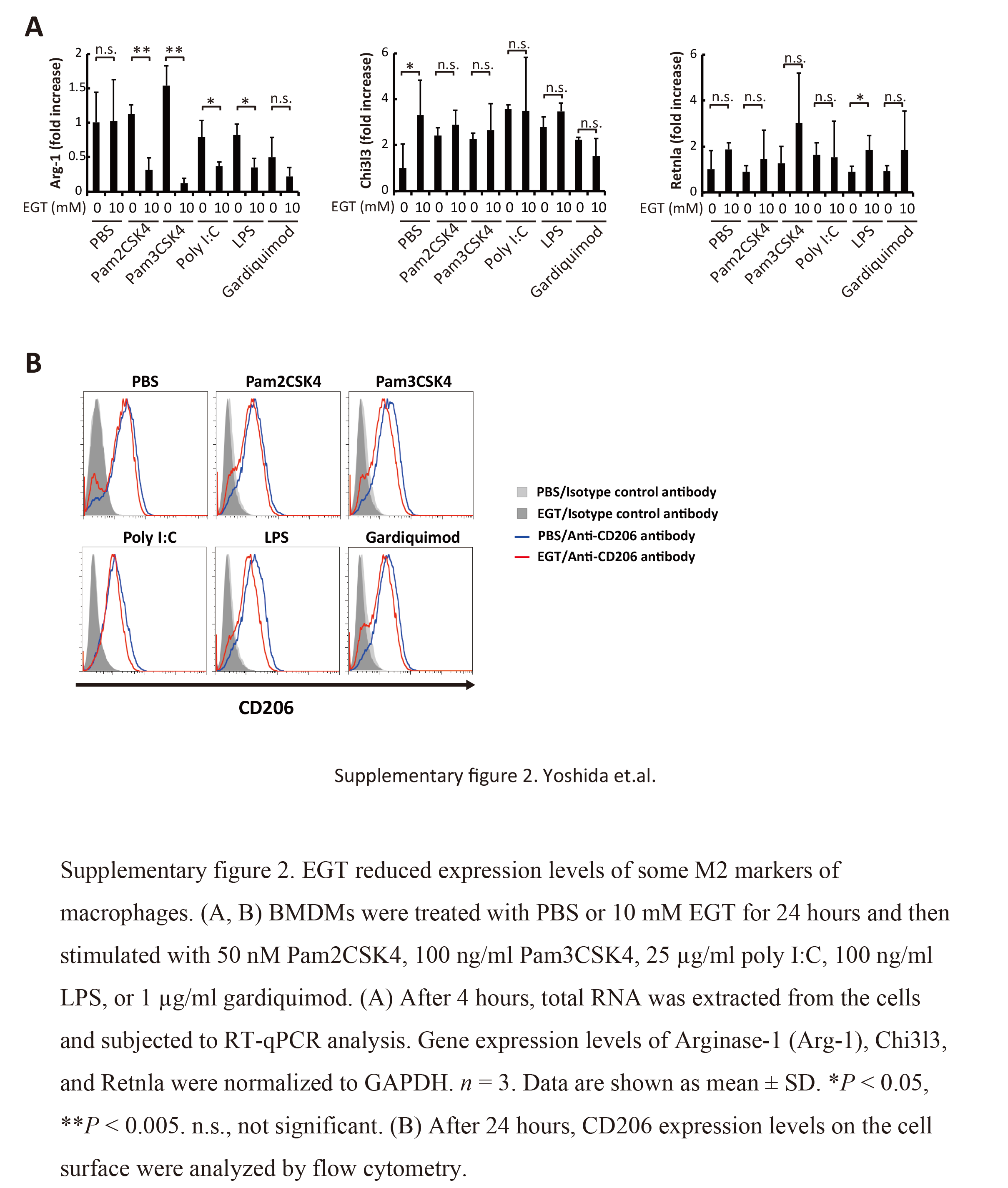

Supplement: S2 Fig — (A) BMDMs were treated with PBS or EGT for 24 hrs and then stimulated with Pam2CSK4, Pam3CSK4, poly I:C, LPS or gardiquimod. After 4 hrs, total RNA was extracted from the cells and subjected to RT-qPCR. (B) BMDMs were treated with PBS or EGT and then stimulated with the TLR ligands as in panel A. After 24 hrs, CD206 expression levels on the cells were measured by FACS. (TIF) [file pone.0169360.s002.tif]

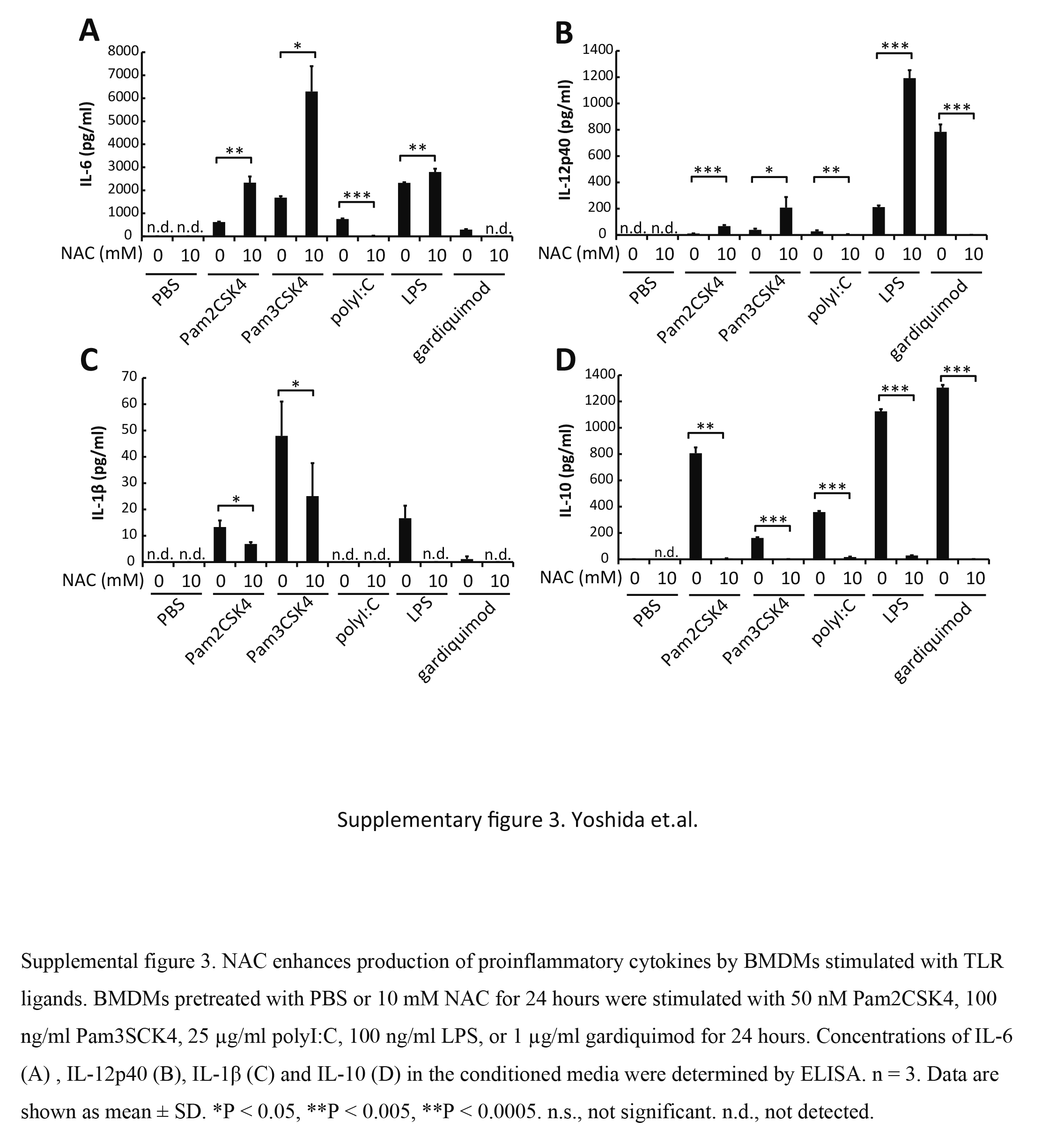

Supplement: S3 Fig — BMDMs were pretreated with PBS or NAC for 24 hrs, and then stimulated with the indicated TLR ligands for 24 hrs. The supernatant levels of (A) IL-6, (B) IL-12p40, (C) IL-1β, and (D) IL-10 were determined. (TIF) [file pone.0169360.s003.tif]
